# Association between preoperative radiographic osteoarthritis severity and patient-reported outcomes after total hip arthroplasty: a systematic review and meta-analysis

**DOI:** 10.1007/s00264-026-07000-0

**Published:** 2026-09-16

**Authors:** Junya Yoshitani, Joseph Dulleston, Amir Reza Rafati Fard, Mihir Gajre, Vikas Khanduja

**Affiliations:** 1 Young Adult Hip Service, Department of Trauma and Orthopaedics, Cambridge University Hospitals NHS Foundation Trust, Cambridge, United Kingdom; 2 Department of Orthopaedics, Toyama General Hospital, Toyama, Japan; 3 School of Clinical Medicine, University of Cambridge, Cambridge, United Kingdom; 4 Imperial College Healthcare NHS Trust, London, United Kingdom; 5 Barts Health NHS Trust, London, United Kingdom

**Keywords:** Total hip arthroplasty, Osteoarthritis, Radiographic severity, Patient-reported outcomes, Systematic review, Meta-analysis

## Abstract

**Purpose:**

Although total hip arthroplasty (THA) reliably improves pain and function in patients with hip osteoarthritis (OA), a subset of patients experience limited postoperative benefit. The association between preoperative radiographic OA severity and postoperative patient-reported outcomes (PROMs) remains controversial.

**Methods:**

We performed a systematic review and meta-analysis according to PRISMA guidelines. Electronic databases (MEDLINE, Embase, and Cochrane Library) were searched for studies comparing postoperative PROMs after primary THA between patients with moderate versus severe radiographic OA. The primary outcome was postoperative PROMs at approximately 12 ± six months. Random-effects meta-analyses were conducted using standardized mean differences (SMDs). Adjusted continuous and responder outcomes, complications, and sensitivity analyses were also evaluated.

**Results:**

Eighteen observational studies (9,235 patients) were included. Severe radiographic OA showed statistically significant but modest advantages in function (SMD − 0.34; 95% CI − 0.62 to − 0.07; I^2^ = 83%) and health-related quality of life (HRQoL; SMD − 0.33; 95% CI − 0.54 to − 0.11; I^2^ = 70%), while pain did not differ (SMD − 0.14; 95% CI − 0.51 to 0.22; I^2^ = 79%). Substantial heterogeneity limits confidence in these estimates. Adjusted analyses showed a mean difference of − 4.0 points on function scales and 49% lower odds of a clinically meaningful response in the moderate group (OR 0.51; 95% CI 0.36–0.72). No statistically detectable differences in complication rates were observed.

**Conclusion:**

Greater preoperative radiographic OA severity was associated with statistically significant but modest advantages in postoperative function and HRQoL, whereas pain relief was comparable across severity groups. Although patients with moderate OA may experience symptomatic improvement, their likelihood of achieving a clinically meaningful response appears lower. These findings support careful patient selection, realistic expectation-setting, and thorough evaluation of pain sources before proceeding to THA.

**Supplementary Information:**

The online version contains supplementary material available at https://doi.org/10.1007/s00264-026-07000-0.

## Introduction

Total hip arthroplasty (THA) is one of the most successful and cost-effective procedures for treating hip osteoarthritis (OA), substantially improving patients’ quality of life [1, 2]. However, a subset of patients experience limited pain relief and functional improvement after surgery [3–6]. Identifying predictors of postoperative outcomes is therefore essential to support shared decision-making. Patient-reported outcome measures (PROMs) are commonly employed to evaluate the achievement of fundamental objectives in THA, and they provide a valuable means for incorporating patient-centered perspectives into the assessment of value [7, 8]. Several predictors of poor PROMs have been investigated, such as age, sex, comorbidities and previous surgery [9–13]. Preoperative radiographic severity of OA has been evaluated as a predictor of postoperative improvement. The published evidence is markedly conflicting. Several cohort studies have reported no association between preoperative radiographic grade and postoperative PROMs, concluding that the preoperative radiograph is of limited prognostic value. [14–16]. In contrast, other investigators have found that patients with milder radiographic change achieve inferior postoperative function and are less likely to reach a clinically meaningful response [17, 18]. These discrepancies are difficult to reconcile because prior studies have been limited by small sample sizes, variable grading systems, and inconsistent outcome reporting. The critical gap in the literature is therefore the absence of a quantitative synthesis estimating the magnitude of this association across function, pain, and health-related quality of life (HRQoL) simultaneously.

The objective of this systematic review and meta-analysis was to quantify the association between preoperative radiographic OA severity and postoperative PROMs following primary THA, and to determine whether patients with moderate radiographic OA achieve inferior outcomes compared with those with severe disease.

The primary research question was: Do patients with moderate radiographic OA achieve inferior postoperative PROMs after THA compared with those with severe OA?

We hypothesised that patients undergoing primary THA for moderate radiographic OA would achieve inferior postoperative PROMs compared with those with severe OA. This expectation was grounded in the premise that the radiographic grade captures only the structural component of the disease: in patients with milder radiographic change, a greater proportion of symptoms may arise from non-articular pain generators—such as spinal, periarticular, or soft-tissue pathology—that are not corrected by arthroplasty. Plain radiographic grading may also underestimate the true structural burden in some patients, further weakening the correspondence between radiographic severity and the symptoms that arthroplasty is able to relieve.

## Methods

### Protocol and registration

This study was conducted according to the Cochrane Handbook for Systematic Reviews, and reported as per the Preferred Reporting Items for Systematic Reviews and Meta-Analyses 2020 (PRISMA) guidelines [19]. This systematic review was registered in PROSPERO (Reg no: CRD42023445918). This systematic review did not require ethical approval as it involved only secondary analysis of previously published data. Human and animal rights were not applicable to this study.

### Eligibility criteria

We included English-language, original studies (retrospective or prospective cohorts and RCTs) of adult patients (> 18 years) undergoing primary THA for primary or secondary osteoarthritis, in which postoperative PROMs were reported in relation to preoperative radiographic severity. We adopted each study's own dichotomisation, with comparisons structured as moderate (Kellgren–Lawrence or Tönnis grades 0–2 or 1–2) versus severe (grades 3–4). We excluded studies lacking preoperative OA grade or joint-space-width information, studies not evaluating the grade–PROM relationship, non-original studies, revision, resurfacing and hemiarthroplasty, duplicate cohorts, and abstract-only reports. The restriction to English-language publications was pragmatic; although it may introduce language bias, the majority of high-quality arthroplasty registry and cohort studies are published in English, and this limitation is acknowledged below. Detailed information is provided in Supplementary Material 1.

### Information sources and search strategy

MEDLINE (via PubMed), Embase, and the Cochrane Library were searched from inception to 24 March 2023, with an updated search on 12 October 2025, by two reviewers. Search terms combined three concepts: hip arthroplasty (e.g. "hip replacement", "hip arthroplasty", "hip prosthesis"); hip osteoarthritis and radiographic severity (e.g. "osteoarthritis, hip", "radiographic severity", "joint space", "Kellgren-Lawrence", "Tönnis", "Croft", "grade", "stage"); and outcomes (e.g. "outcome assessment", "patient-reported outcome"). No date, design, or geographical restrictions were applied. Reference lists of included studies were also screened. Full search strategies with record counts are provided in Supplementary Material 1.

### Selection process

Identified articles from electronic database searching were imported into Endnote 20 (Clarivate Analytics) and Rayyan (Rayyan Systems) and deduplicated. Articles were screened by title and abstracts according to the eligibility criteria by two independent reviewers, followed by full-text screening. Any discrepancies were discussed in a consensus meeting, and if necessary, resolved by a third reviewer.

### Data collection process and data items

Two reviewers independently extracted data using pre-piloted Excel spreadsheets. Extracted data included study and population characteristics, radiographic severity measures and thresholds, and PROMs, including Western Ontario and McMaster Universities Arthritis Index (WOMAC), Hip disability and OA Outcome Score (HOOS), Oxford hip score (OHS), EuroQol-5 Dimension (EQ-5D), the 36-Item Short Form Health Survey (SF-36) (all available subscales), Veterans RAND 12-Item Health Survey (VR-12), and patient satisfaction. Complications and other reported outcomes were also recorded when available.

Several analytic decisions were prespecified. The primary time point of interest was 12 ±  six months (range, 6 to 18 months). When multiple eligible time points were reported, we selected outcomes closest to 12 months; if more than one remained, the time point with the least missing data was chosen, followed by the longest available follow-up. For each analysis, outcomes were prioritized hierarchically, with unadjusted postoperative between-group differences (group means and standard deviations) considered primary, and adjusted between-group estimates (ANCOVA-adjusted postoperative differences or adjusted differences in change scores) considered secondary.

To avoid double counting, only one PROM per construct was included per study. For functional outcomes, the prespecified priority order was HOOS–Activities of Daily Living (ADL), WOMAC-Function, OHS, and SF-36 Physical Function. For pain outcomes, the priority order was HOOS-Pain, WOMAC-Pain, and, as auxiliary measures, pain visual analog or numeric rating scales. For health-related quality of life (HRQoL), EQ-5D index scores were prioritized, followed by SF-36 or VR-12 Physical Component Summary scores. When multiple eligible instruments were reported at the same priority level, selection was based on the authors’ designated primary outcome, followed by the extent of missing data and the most widely used instrument.

All outcome scales were oriented so that higher scores indicated better status whenever possible. Effect sizes were coded as mean differences calculated as moderate minus severe radiographic severity, such that positive values favored the moderate severity group. Missing standard deviations (SDs) were derived from available summary statistics when possible. The level of evidence in each study was determined in accordance with the updated JBJS-Am level of evidence criteria [20].

### Risk of bias assessment

Assessment of the risk of bias was carried out independently by two reviewers using the Cochrane Risk-of-Bias (RoB) 2.0 Assessment tool for randomized trials [21], and Cochrane’s ROBINS-I tools were used for non-randomized trials containing seven domains [22]. Each domain and overall risk were categorized as low, some concerns, serious/high, or critical.

### Effect measures and synthesis of results

Summary statistics were reported as means with SDs or 95% confidence intervals (95% CIs). Where appropriate, outcomes were pooled across studies. Differences in outcomes between the moderate and severe arthritis groups were visually presented using forest plots.

For clinical outcomes expressed as continuous variables, meta-analyses were performed using RevMan 5.4. Overall mean differences (MDs) or standardized mean differences (SMDs) for preoperative and postoperative PROMs were calculated with corresponding 95% CIs, using a random-effects model with the inverse-variance method.

For the primary synthesis, unadjusted postoperative between-group differences were pooled. When outcome measures were assessed using the same instrument, results were synthesized as MDs; when different instruments were used to measure the same construct, results were synthesized as SMDs (Hedges’ g).

For the secondary synthesis, adjusted between-group effect estimates were pooled using the Generic Inverse Variance method by entering the effect estimate and its standard error (SE).

For dichotomous responder outcomes, the event was defined as being a “responder.” When adjusted odds ratios (ORs) were reported, log-transformed ORs were pooled using the Generic Inverse Variance method. When adjusted estimates were unavailable, ORs were calculated from 2 × 2 contingency tables. If studies reported ORs for non-responders, these were inverted to derive responder ORs, with corresponding reversal of the log scale.

Statistical heterogeneity was quantified using the I^2^ and τ^2^ statistics and was explored through pre-specified subgroup and sensitivity analyses, including severity definition, follow-up time point, age group, and aetiology.

### Additional analyses

Additional analyses were performed to further explore the robustness and clinical relevance of the findings. First, unadjusted responder analyses were conducted for studies reporting dichotomous responder outcomes. Responders were defined according to study-specific criteria, including patient acceptable symptom state (PASS), substantial clinical benefit (SCB), and minimal clinically important difference (MCID).

Second, complication outcomes were synthesized descriptively and, where data permitted, quantitatively.

Third, sensitivity analyses were performed to assess the robustness of the primary findings. Sensitivity analyses included leave-one-out analyses, alternative meta-analytic models, exclusion of studies requiring statistical conversion of summary data, and removal of influential studies identified a priori. Details of these sensitivity analyses and their results are provided in Supplementary material 2.

The certainty of evidence was not formally graded using GRADE; instead, robustness was evaluated through risk-of-bias assessment and prespecified sensitivity analyses.

### Difference between protocol and review

This review followed the prespecified PROSPERO protocol (CRD42023445918), with minor methodological deviations.

Because of heterogeneity in adjustment strategies and covariates, unadjusted postoperative between-group differences were prioritized as the primary analyses, with adjusted estimates analyzed secondarily.

Given variability in outcome instruments, construct-based syntheses (function, pain, HRQoL) using standardized mean differences and additional sensitivity analyses were performed to enable scale-agnostic comparisons.

We also conducted supplementary unadjusted responder analyses (PASS, SCB, MCID) and descriptive complication analyses not explicitly prespecified.

These deviations were methodologically justified to enhance robustness and clinical interpretability and are unlikely to have materially affected the study conclusions.

## Results

### Study selection

**Fig. 1:**
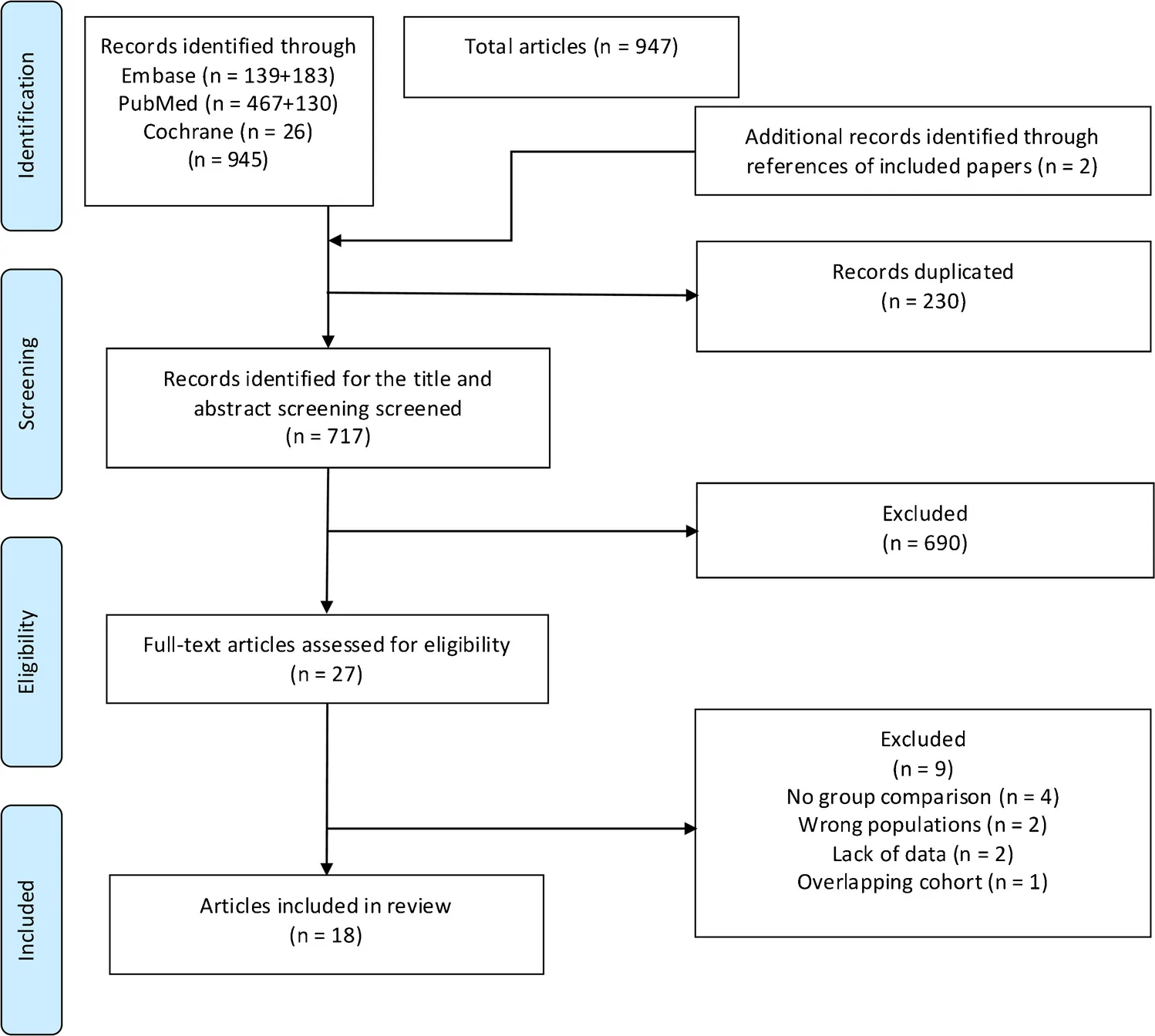
The algorithm for selecting articles from databases

Electronic database searching yielded 945 records. In addition, two studies were identified through citation searching of articles included in the initial systematic search. After removal of duplicates, 717 records were screened by title and abstract, followed by full-text assessment of 27 articles. Of these, 18 studies met the inclusion criteria and were included in the final analysis (Fig. 1) [10, 15, 17, 23–36].

### Patient characteristics and quality of included studies

Study and patient characteristics are summarized in Table 1. Across all cohorts, 9,235 participants were included. All studies were observational in design. Radiographic severity was operationalized using Kellgren–Lawrence or Tönnis grading systems, with most comparisons structured as moderate (grades 0–2 or 1–2) versus severe (grades 3–4) OA at approximately 12 ± six months after primary THA. PROMs spanned hip-specific (e.g., WOMAC, OHS, HOOS) and generic health instruments (e.g., EQ-5D, SF-36/VR-12), with outcome availability and time points varying by study.

**Table 1:** Study characteristics and patient demographics

Study	Year	Design	Sample size (hips)	Age, mean	Follow-up, month	Etiology	Severity instrument	Moderate definition	Severe definition	Primary Function PROM	Pain	HRQoL	Level of Evidence
Tilbury	2016	Prospective cohort	302	moderate: 66 severe: 68	12	OA	KL	0–2	3–4	HOOS ADL	HOOS pain	EQ-5D	II
Al-Amiry	2017	Prospective cohort	222	68	12	Primary OA	KL	0–2	3–4	WOMAC-Function	NR	EQ-5D	II
Nilsdotter	2001	Prospective cohort	162	71.2	12	Primary OA	OARSI criteria	2	3	WOMAC-Function	WOMAC-Pain	SF-36	II
Gorur	2025	Retrospective cohort	222	moderate: 59 severe: 70	12	Mild to moderate vs Advanced OA	KL	1–3, labral tear	4	WOMAC-Function	WOMAC-Pain	SF-12 M	III
Neitzke	2024	Retrospective cohort	371	moderate: 65 severe: 63	12	OA	KL	0–2	4	HOOS-JR	VAS	VR-12	III
Peeters	2024	Retrospective case-control study	100	56	12	OA	Tönnis	1	3	HOOS-ADL	HOOS pain	SF-36	III
Sharrock	2024	Retrospective cohort	270	moderate: 61 severe: 66	12	Primary OA	KL	0–2	3–4	OHS	NR	EQ-5D	III
Kashanian	2025	Retrospective cohort	694	64.5	12	OA	Tönnis	2	3	OHS	NR	EQ-5D-5L	III
Aggarwal	2022	Registry-based cohort	1412	67	6	Primary OA	KL JSN/osteophyte/sclerosis	0–2	3–4	OHS	NR	Satisfaction	III
Dowsey	2016	Prospective cohort	382	68.9	12–24	Primary THA	mKL	< 4	4	HHS pain/function		SF-12	II
Dowsey	2022	Registry-based cohort	2177	66.5	12	Primary THA	KL	< 4	4	WOMAC-Function	WOMAC-Pain	VR-12	III
Judge	2011	Cohort	845	NR	12	Primary THA	KL grade/JSW	0–3	4	OMERACT-OARSI (WOMAC-based function)	WOMAC	EQ-5D	III
Keurentjes	2013	Prospective cohort (questionnaire follow-up)	445	66.6	33.6 (11.16)	Primary THA	KL	0–2	3–4	SF-36	NR	SF-36	II
Neuprez	2020	Prospective cohort (hip n = 346	346	64.6	12–60	End-stage Hip OA	KL	0–2	3–4	WOMAC-Function	WOMAC-Pain	SF-36	II
Rojanasopondist	2019	Prospective data analysed retrospectively	594	60	13 months	Primary OA	JSW (mm)	NR	NR	SF-36	NRS Pain	EQ-5D	II
Stambough	2016	Retrospective cohort (≤ 55y)	146	43.2	24 months	Primary OA	JSW (mm)	NR	NR	mHHS	WOMAC Pain	SF-12 PCS/MCS	III
Treu	2024	Retrospective cohort	263	47	4.4 years	Dysplasia THA	Tönnis	1–2	3	HOOS JR	PROMIS Pain	PROMIS Physical/Mental	III
Cushnaghan	2007	Prospective longitudinal cohort + population controls	282	76.3	8.5 years	Primary OA	Croft grade	0–3 or 4	5	SF-36	NR	SF-36	II

*HRQoL* Health-Related Quality of Life, *OARSI* Osteoarthritis Research Society International, *PROMs* Patient-reported outcome measures, *OA* Osteoarthritis, *HOOS* Hip disability and Osteoarthritis Outcome Score, *SF-36* the 36-Item Short Form Health Survey, *HHS* Harris hip score, *WOMAC* Western Ontario and McMaster Universities Arthritis Index, *EQ-5D* EuroQol-5 Dimension, *OHS* Oxford hip score, *KL* Kellgren–Lawrence grade, *NRS* Numeric Rating Scale, *NR* Not reported, *JSW* Joint Space Width, *JSN* Joint Space Narrowing, *mKL* modified Kellgren–Lawrence grade, *PROMIS* Patient-Reported Outcomes Measurement Information System, *VR-12* Veterans RAND 12-Item Health Survey, *VAS* Visual Analogue Scale, *SF-12* the 12-Item Short Form Health Survey, *mHHS* modified Harris hip score

### Risk of bias

**Fig. 2:**
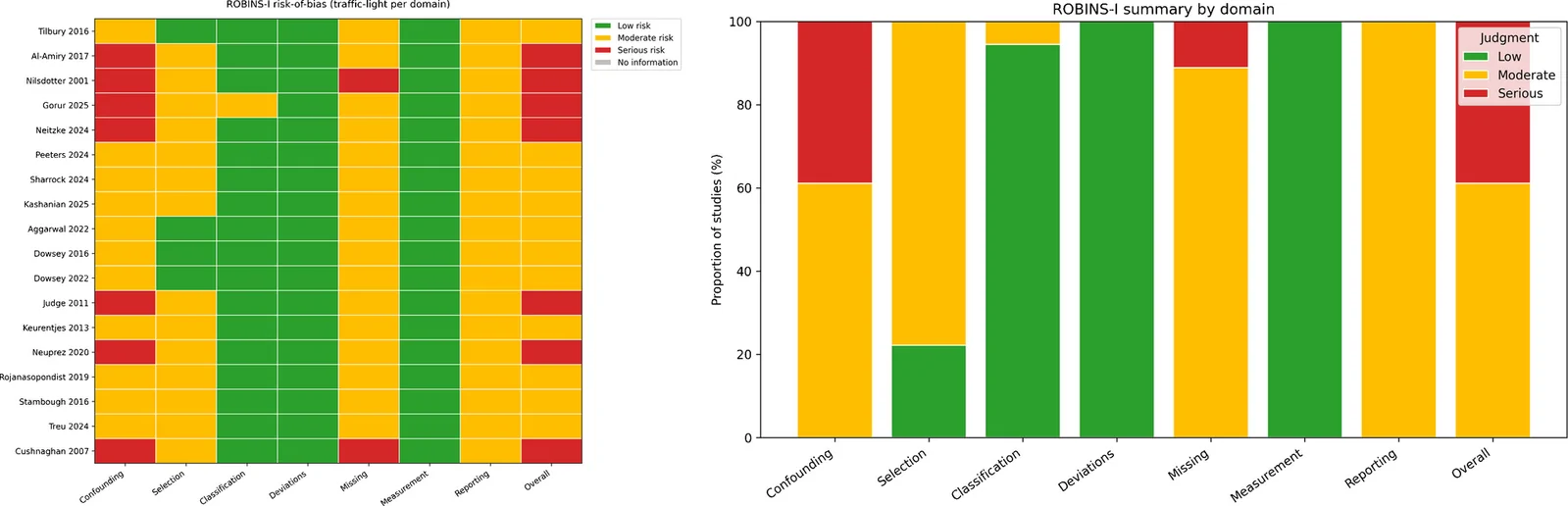
Risk of Bias of non-randomized studies using ROBINS-I

Risk of bias was assessed using ROBINS-I for nonrandomized studies. Most cohorts were judged to have an overall moderate risk of bias, primarily due to potential confounding, missing outcome data, and selective reporting of PROM subscales. Several older or unadjusted cohorts were rated at serious overall risk, largely because group comparisons were unadjusted and/or attrition was substantial. Domains relating to classification of interventions and deviations from intended interventions were generally low risk. As a robustness check, restricting meta-analyses to studies with overall risk ≤ moderate produced directionally consistent estimates and unchanged qualitative conclusions. Full domain-level ratings are presented in Fig. 2.

### Primary analysis

**Fig. 3:**
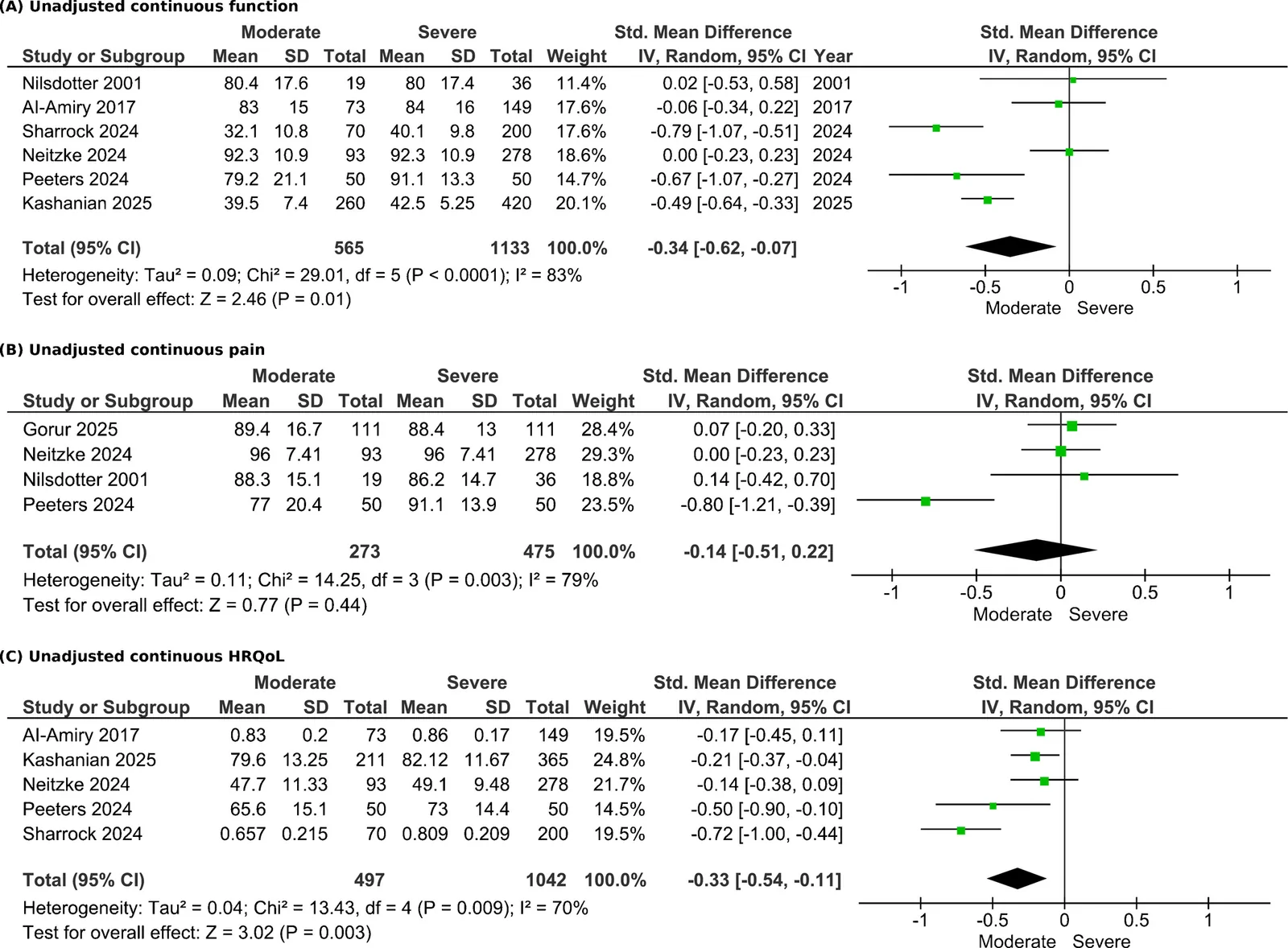
Primary unadjusted postoperative PROMs at 12 ± 6 months. Forest plots showing the primary unadjusted postoperative patient-reported outcome measures (PROMs) comparing patients with moderate versus severe radiographic osteoarthritis at approximately 12 ± 6 months after primary total hip arthroplasty. **A** Function outcomes, **B** pain outcomes, and **C** health-related quality of life (HRQoL) outcomes are presented as standardized mean differences (SMDs) with 95% confidence intervals (CIs), synthesized using random-effects models. Negative values indicate better outcomes in the severe osteoarthritis group

Function: Across six studies, patients with severe radiographic OA showed statistically significantly better postoperative function than those with moderate OA (pooled SMD = –0.34, 95% CI –0.62 to –0.07; I^2^ = 83%), corresponding to a small effect by conventional criteria (Fig. 3A).

Pain: Four studies showed no clear difference in postoperative pain between groups (pooled SMD = –0.14, 95% CI –0.51 to 0.22; I^2^ = 79%) (Fig. 3B).

HRQoL: Five studies indicated a statistically significant advantage of small magnitude for the severe OA group (pooled SMD = –0.33, 95% CI –0.54 to –0.11; I^2^ = 70%) (Fig. 3C).

### Sub analysis

#### Adjusted continuous

**Fig. 4:**
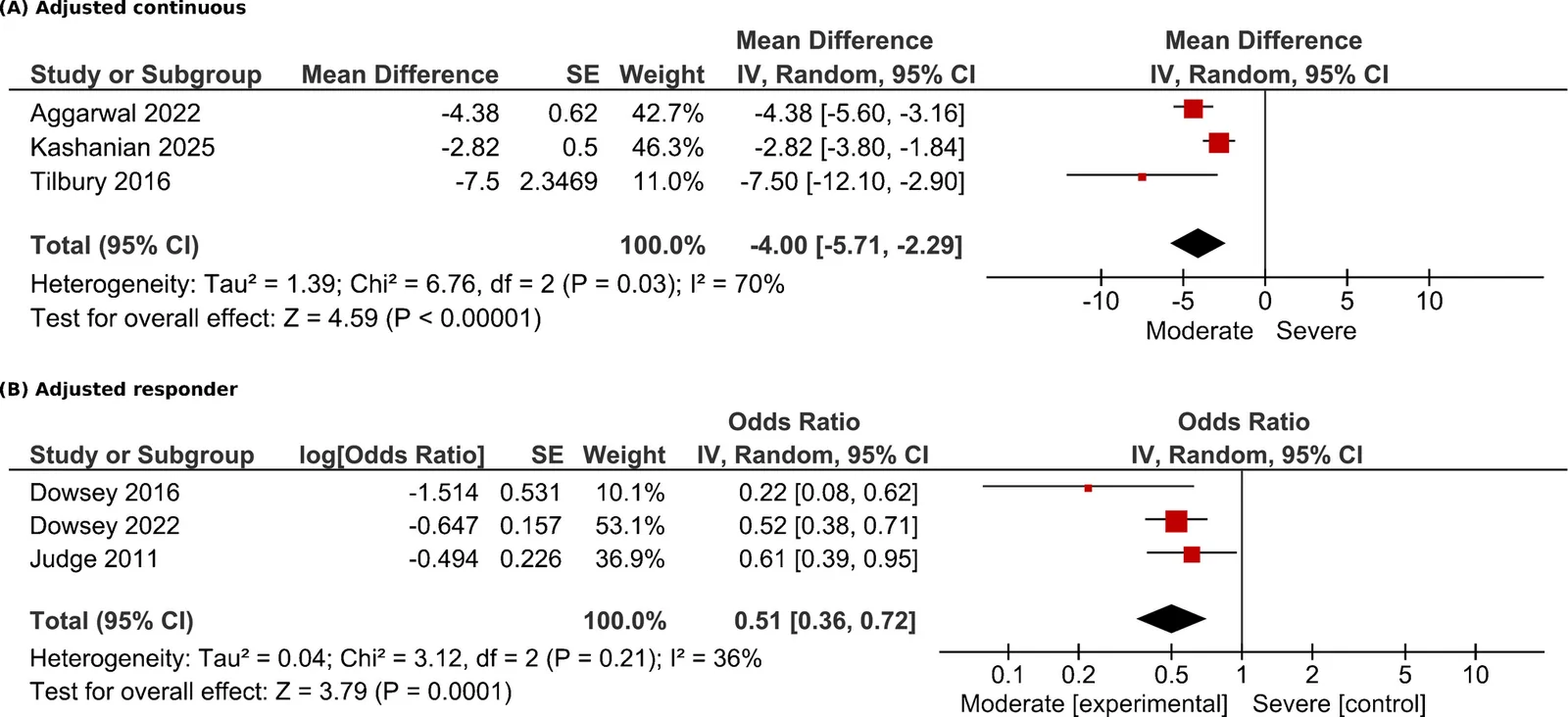
Adjusted postoperative outcomes. Forest plots summarizing adjusted postoperative outcomes comparing moderate versus severe radiographic osteoarthritis. **A** Adjusted continuous outcomes pooled as mean differences (MDs) using the Generic Inverse Variance method, based on multivariable-adjusted estimates reported by individual studies. **B** Adjusted responder outcomes pooled as odds ratios (ORs) with 95% confidence intervals, where the event was defined as achieving a clinically meaningful response according to study-specific criteria. Values < 1 indicate lower odds of response in the moderate osteoarthritis group. Random-effects models were applied in all analyses

Three studies reporting adjusted between-group differences on 0–100 scales (OHS/HOOS-ADL) were pooled using a generic inverse-variance random-effects model (15, 32, 37). The pooled adjusted MD was − 4.00 [95% CI − 5.71 to − 2.29], with substantial heterogeneity (I^2^ = 70%). Thus, after covariate adjustment at ~ six to 12 months, the Severe OA group scored on average ~ 4 points better than the Moderate group on function scales (Fig. 4A).

### Adjusted responder

Across three studies using multivariable logistic models at ~ 12 months [25, 26, 32], patients with less-severe radiographic OA had lower adjusted odds of being a responder compared with those with severe OA: pooled OR = 0.51 (95% CI 0.36–0.72); *I*^2^ = 36% (random-effects) (Fig. 4B). Responder definitions were OMERACT-OARSI composite response [26, 32] and HHS MID attainment [25]. Covariate adjustment included age, sex, BMI, baseline PROMs, ASA/comorbidity measures and other study-specific factors. Results indicate ~ 49% lower adjusted odds of achieving a clinically meaningful response in the moderate group versus severe OA at one year. Gorur 2025 was not included in this adjusted-responder meta-analysis because only domain-specific odds ratios were reported and the WOMAC-Function CI contained an internal inconsistency that precluded a reliable SE derivation [27]; Keurentjes 2013 was analyzed as supplementary due to longer follow-up and HRQoL domain focus [17] (Supplementary material 2).

### Complications

Pooled analyses showed no statistically detectable differences in dislocation, superficial infection, or periprosthetic fracture between severity groups; however, event counts were very low and these analyses were underpowered to establish equivalence ADDIN EN.CITE [28–31] (Supplementary material 2).

### Studies not eligible for the primary meta-analysis

Five studies did not provide extractable unadjusted 12 ± six month postoperative means and SDs for a dichotomous radiographic severity comparison and were therefore excluded from the primary quantitative synthesis [10, 24, 33–35] **(**Supplementary material 2**)**.

Three studies using joint-space width or Croft grade consistently reported greater functional improvement in more severe OA, whereas pain and HRQoL findings were limited and inconsistent [10, 24, 35]. Two additional studies were not comparable because severity stratification was unavailable or etiologically distinct [33, 34]. Overall, non-pooled evidence aligned directionally with the primary analyses but was limited by indirectness and imprecision.

### Sensitivity analyses

Sensitivity analyses yielded results that were largely consistent with the primary analyses. For functional outcomes, the direction of effect was robust across leave-one-out analyses, alternative models, and exclusion of influential cohorts, although the magnitude of the pooled effect was attenuated when large or high-leverage studies were removed. Instrument-restricted analyses showed no material difference when restricted to WOMAC-Function, whereas OHS-based analyses favored the Severe group with moderate heterogeneity.

For pain outcomes, no overall difference between severity groups was observed. Analyses restricted to WOMAC-Pain demonstrated no association with radiographic severity (SMD 0.08, 95% CI − 0.16 to 0.32; I^2^ = 0%), while non-WOMAC instruments showed heterogeneous and non-significant results.

For HRQoL, EQ-5D–based analyses favoured the Severe group (SMD − 0.36, 95% CI − 0.68 to − 0.03), whereas non–EQ-5D instruments demonstrated a similar but non-significant trend. Because fewer than ten studies contributed to each primary meta-analysis, we did not formally assess publication bias or small-study effects, as such methods are underpowered in this setting.

## Discussion

In this systematic review and meta-analysis of patients undergoing primary THA, more severe preoperative radiographic OA was associated in a directionally consistent pattern with equal or superior postoperative outcomes compared with moderate radiographic disease; however, the magnitude of effect was modest and the high heterogeneity (I^2^ = 70–83%) limits confidence in the pooled estimates. In unadjusted analyses at approximately 12 months, patients with severe OA demonstrated slightly better postoperative function (SMD − 0.34) and HRQoL (SMD − 0.33), while pain outcomes did not differ meaningfully between severity groups. These effect sizes are near or below the commonly cited minimum important difference thresholds for hip-specific PROMs, and statistical significance should not be equated with clinical importance. These directional trends were supported—but not definitively confirmed—by adjusted analyses based on only three studies each, in which severe OA was associated with better functional scores and higher odds of achieving clinically meaningful improvement. Complication rates were low and did not differ materially by radiographic severity. Sensitivity analyses yielded directionally consistent results.

These findings are consistent with prior reports suggesting that patients with low-to-moderate radiographic OA may achieve less favorable functional outcomes after THA despite experiencing pain relief. [29–31, 37].

A plausible explanation is the discordance between radiographic OA severity and symptom aetiology. Favorable THA outcomes in patients labeled as having “early OA” on plain radiographs are largely confined to those with occult advanced structural changes, such as severe joint-space narrowing or subchondral cysts identified on CT or MRI [30, 37]. In contrast, patients without such structural pathology may have symptoms influenced by multifactorial and non-OA pain generators, including femoroacetabular impingement, periarticular soft-tissue pain, spinal pathology, or psychosocial factors, which are not fully corrected by arthroplasty [29, 31].

Although function and HRQoL tended to favour the Severe group, pain did not show a clear between-group difference. Persistent postoperative pain in patients with lower radiographic severity may reflect non-articular pain generators or altered pain processing rather than residual joint pathology [16, 18]. In addition, pain instruments may be less sensitive to severity-related differences once the primary nociceptive source is removed, which may explain the attenuated and instrument-dependent pain effects observed in sensitivity analyses. This dissociation between pain and function is mechanistically plausible. Arthroplasty reliably removes the intra-articular nociceptive source, so pain relief is achieved largely irrespective of preoperative radiographic grade. Restoration of function, by contrast, may depend additionally on factors such as periarticular soft-tissue condition and concurrent spinal or extra-articular pathology that are not captured by the radiographic grade. These mechanisms cannot be confirmed within the present dataset and should be regarded as hypotheses for future investigation.

From a clinical perspective, although patients with moderate radiographic OA often experience symptomatic improvement after THA, they appear less likely to achieve superior functional outcomes or clinically meaningful improvement compared with patients with severe OA. These findings support more cautious shared decision-making in this population. Because THA is an elective procedure associated with surgical risk and cost, patients with moderate OA should be informed that outcomes comparable to end-stage disease are not uniformly guaranteed. In patients with moderate OA, the aetiology of pain may not be confined solely to intra-articular osteoarthritic pathology. Reassessment of pain sources—including extra-articular pathology and pain comorbidities—should be considered when imaging and symptoms are discordant [16, 37]. Advanced imaging and diagnostic intra-articular injections may aid in patient selection. Further research using standardised radiographic severity definitions and longer-term follow-up is needed to clarify whether the modest differences observed at approximately one year persist over time.

Furthermore, randomized trials have shown that hip arthroscopy improves HRQoL in patients with femoroacetabular impingement and Tönnis grade 1 OA ADDIN EN.CITE (41, 42). It is important to note that this review did not directly compare THA against joint-preserving or nonoperative alternatives; the finding that patients with moderate OA achieve less improvement after THA does not establish that such patients are candidates for arthroscopy. Rather, these data suggest that before proceeding to THA in patients without end-stage radiographic disease, a thorough evaluation of the pain source and a discussion of all available options is warranted, particularly in younger patients.

### Strengths

This review prespecified the key comparison and 12-month time window and used a construct-based synthesis to address heterogeneous PROM instruments. We incorporated both unadjusted and adjusted analyses and performed prespecified sensitivity analyses　including leave-one-out and influential-study checks, enhancing robustness and interpretability in a heterogeneous literature.

### Limitations

All included studies were observational, with potential residual confounding and variable adjustment strategies. Heterogeneity across radiographic definitions, PROM instruments, and patient populations was substantial. The primary synthesis focused on postoperative status at 12 months rather than change scores, and reporting of complications and responder definitions was inconsistent. Publication bias could not be formally assessed due to the limited number of studies per outcome. A key qualitative limitation is the heterogeneity in terminology: the definition of “moderate” OA varied substantially across studies (e.g., Kellgren–Lawrence grade 1–2 in some versus grade 2 alone in others; Tönnis grade 1 versus ≥ 1), meaning that what one study classified as “moderate” may correspond to “mild” or even “severe” in another. This is a fundamental source of heterogeneity that limits cross-study comparisons. The restriction to English-language publications may have introduced language bias. Finally, adjusted secondary analyses were based on only three studies each with differing covariate sets and should be regarded as supportive rather than confirmatory.

## Conclusion

In patients undergoing primary THA, more severe preoperative radiographic OA was associated with statistically significant but small advantages in postoperative function and HRQoL at approximately 12 months, whereas pain relief was comparable across severity groups. Although patients with moderate radiographic OA can experience meaningful symptomatic improvement after arthroplasty, their likelihood of achieving a clinically meaningful response appears lower than that of patients with end-stage disease. These findings underscore the importance of careful patient selection and shared decision-making, particularly in patients with moderate OA or in younger individuals. Prospective studies with standardised severity definitions and longer follow-up are needed to determine whether these differences persist beyond the first postoperative year.

## Supplementary Information

Below is the link to the electronic supplementary material.

ESM 1 (DOCX 32.9 KB)

ESM 2 (DOCX 25.8 KB)

ESM 3  (DOCX 84.0 KB)

## Data Availability

No datasets were generated or analysed during the current study.
