# Supplementary material for: Association between preoperative radiographic osteoarthritis severity and patient-reported outcomes after total hip arthroplasty: a systematic review and meta-analysis
Source: Int Orthop. 2026 Sep 16;50(11):2895–906. doi: 10.1007/s00264-026-07000-0 (PMC13694559; doi:10.1007/s00264-026-07000-0)
Supplement: Supplementary file 2 [file 264_2026_7000_MOESM2_ESM.docx]

**Eligibility Criteria**

Inclusion criteria

Adult patients (age > 18 years) who undergo primary THA due to primary or secondary osteoarthritis

Study with post-operative PROMs after THA

Original studies including retrospective, prospective cohort studies and RCTs

Studies reported in the English language

Exclusion criteria

Study without information about preoperative osteoarthritis grade or joint space width

Study without evaluation between arthritis grade and post-operative PROMs

Animal or cadaveric studies

Studies that were not original (e.g. systematic reviews, narrative reviews, technical notes)

Patients who undergo revision hip arthroplasty, resurfacing hip arthroplasty and hemiarthroplasty

Duplicate cohorts

Only abstract study

**Searching strategy**

| MEDLINE (Pubmed) | Search Details | Results | |
| --- | --- | --- | --- |
|  | ("arthroplasty, replacement, hip"[MeSH Terms] OR "hip replacement"[Title/Abstract] OR "hip arthroplasty"[Title/Abstract] OR "hip prosthesis"[Title/Abstract]) AND ("hip Osteoarthritis"[Title/Abstract] OR "osteoarthritis, hip"[MeSH Terms] OR "hip-related pain"[Title/Abstract] OR "hip pain"[Title/Abstract]) AND ("mild arthritis"[Title/Abstract] OR "radiographic severity"[Title/Abstract] OR "joint space"[Title/Abstract] OR "Tonnis"[Title/Abstract] OR "radiographic change"[All Fields] OR "Kellgren-Lawrence"[Title/Abstract] OR "KL"[Title/Abstract] OR "grade"[Title/Abstract] OR "stage"[Title/Abstract]) AND ("outcome"[All Fields] OR "outcomes"[All Fields]) | 467+130 | |
| EMBASE | Query | Results | |
| 1 | total hip arthroplasty.mp. or exp total hip replacement/ | 32,293 | |
| 2 | hip osteoarthritis.mp. or exp hip osteoarthritis/ | 15,187 | |
| 3 | tonnis grade.mp. | 535 | |
| 4 | joint space.mp. | 9,458 | |
| 5 | kellgren lawrence.mp. | 5,748 | |
| 6 | croft.mp. | 190 | |
| 7 | grade.mp. | 707,830 | |
| 8 | exp outcome assessment/ or exp patient-reported outcome/ or exp clinical outcome/ or outcome.mp. | 3,404,075 | |
| 9 | 1 and 2 | 3,543 | |
| 10 | 3 or 4 or 5 or 6 or 7 | 717,700 | |
| 11 | 8 and 9 and 10 | 139 | |
| EMBASE (18^th^ Jan 2025) | Query |  | |
| 1 | exp Hip Osteoarthritis/ OR ((hip adj3 osteoarthrit*) OR coxarthros*).ti,ab,kw. | 21605 | |
| 2 | exp Hip Arthroplasty/ OR exp Total Hip Arthroplasty/ OR ((total hip adj2 (arthroplast* OR replacement)) OR (hip adj2 replacement)).ti,ab,kw. | 72603 | |
| 3 | exp Osteoarthritis Severity/ OR exp Joint Space Narrowing/ OR ("joint space width" OR "minimum joint space width" OR (joint space adj3 narrow*) OR JSW).ti,ab,kw. | 5718 | |
| 4 | ("Kellgren Lawrence" OR "Kellgren–Lawrence" OR (KL adj2 grade*)).ti,ab,kw. | 7660 | |
| 5 | ((tonnis OR tönnis) adj2 (grade* OR classification)).ti,ab,kw. | 814 | |
| 6 | (Croft adj3 (grade* OR grading OR index)).ti,ab,kw. | 43 | |
| 7 | ((severity OR grade* OR grading) adj3 (osteoarthrit* OR OA OR coxarthros*)).ti,ab,kw. | 7667 | |
| 8 | 3 OR 4 OR 5 OR 6 OR 7 | 17771 | |
| 9 | exp Patient Reported Outcome/ OR exp Quality of Life/ OR exp Outcome Assessment/ OR exp Patient Satisfaction/ OR ((patient-reported OR PRO OR PROM*) adj2 outcome*).ti,ab,kw. OR (EQ-5D OR OHS OR HOOS OR WOMAC OR HHS OR JHEQ OR PROMIS OR satisfaction OR MCID).ti,ab,kw. | 2163599 | |
| 10 | 1 AND 2 AND 8 AND 9 | 183 | |
| Cochrane library | Search | | Hits |
| #1 | MeSH descriptor: [Arthroplasty, Replacement, Hip] explode all trees | | 2860 |
| #2 | MeSH descriptor: [Osteoarthritis, Hip] explode all trees | | 1403 |
| #3 | radiographic severity | | 1845 |
| #4 | joint space | | 2277 |
| #5 | mild arthritis | | 2149 |
| #6 | severe arthritis | | 4802 |
| #7 | tonnis | | 49 |
| #8 | Kellgren-Lawrence | | 2090 |
| #9 | grade | | 97831 |
| #10 | stage | | 115764 |
| #11 | MeSH descriptor: [Outcome Assessment, Health Care] explode all trees | | 218706 |
| #12 | #3 OR #4 OR #5 OR #6 OR #7 OR #8 OR #9 OR #10 | | 200849 |
| #13 | #1 AND #2 AND #11 AND #12 | | 26 |

The original Embase search was conducted in 2023 using the initial strategy (n = 139).
An updated Embase search was performed in 2025 using an expanded but structurally consistent strategy.
Both datasets were merged, and duplicates were removed prior to screening.

Data items

Items included:

- **Study and population**: setting/country, design, sample size, follow-up duration; etiology (primary/secondary/mixed).
- **Radiographic severity**: instrument and thresholds (e.g., KL 0–2 vs 3–4).
- **PROMs**: WOMAC, HOOS, OHS, HHS, EQ-5D, SF-36 (all subscales where available), VR-12, and satisfaction.
- **Complications** and other outcomes if reported.

**Pre-specified choices**:

- **Primary time point**: **12 ± 6 months** (6–18 months). If multiple time points existed, we selected (i) closest to 12 months, then (ii) least missingness, then (iii) longest follow-up.
- **Outcome hierarchy per analysis**:
  1. **Unadjusted postoperative group differences** (group means/SDs),
  2. **Adjusted between-group estimates** (ANCOVA-adjusted postoperative difference or adjusted difference-in-change),
- **Per-construct single outcome per study**: to avoid double counting, we selected one PROM **per construct** using the following priority:
  1. **Function (primary construct)**: HOOS-ADL → WOMAC-Function → OHS → SF-36 PF.
  2. **Pain**: HOOS-Pain → WOMAC-Pain → (auxiliary) pain VAS/NRS.
  3. **HRQoL/generic**: EQ-5D Index → SF-36 PCS (or VR-12 PCS).
     Ties were resolved by authors’ designated primary outcome → least missingness → most widely used instrument.
- **Orientation and coding**: scales were oriented so that **higher = better** whenever possible. Effect sizes were coded as **MD = (moderate − severe)**; positive values favor moderate severity.
- **Missing statistics**: when SDs were missing, we derived SE/SD from 95% CIs or p-values when available; **SD of change** was required to include change outcomes (we did not impute r unless pre-specified for sensitivity analyses).
