# Supplementary material for: Association between preoperative radiographic osteoarthritis severity and patient-reported outcomes after total hip arthroplasty: a systematic review and meta-analysis
Source: Int Orthop. 2026 Sep 16;50(11):2895–906. doi: 10.1007/s00264-026-07000-0 (PMC13694559; doi:10.1007/s00264-026-07000-0)
Supplement: Supplementary file 3 [file 264_2026_7000_MOESM3_ESM.docx]

**Studies not included in the primary meta-analyses (n=5): design and key results**

| Study (year) | Design/setting | Severity definition | Outcome/timepoint(s) | Why not pooled | Direction* | Notes / adjusted effects |
| --- | --- | --- | --- | --- | --- | --- |
| Cushnaghan 2007 | Controlled longitudinal cohort, UK | Croft radiographic grade (0–5) | SF-36 PF & MH, ≥8 y | Timepoint ≫ primary window; scale not harmonizable with other adjusted models | ↑ (Severe better for function) | Adjusted regression: Croft 5 vs 0–3 ≈ +19.4 PF points improvement; MH neutral |
| Stambough 2016 | Retrospective cohort, USA | Minimum joint-space width (JSW) | Functional PROs (THA), 1 y | Predictive model; reporting not directly convertible to SMD/MD by severity groups | ↑ (Severe better) | Narrower preop JSW predicted larger improvements / higher responder odds |
| Rojanasopondist 2019 | Retrospective single-centre THA cohort | JSW categories | MCID/clinically important improvement (OHS/HHS etc.), ~1 y | Predictor analysis; effect reported as aOR per JSW, not group means | ↑ (Severe better) | Smaller JSW (1–2 mm) associated with higher odds of MCID |
| Neuprez 2020 | Prospective registry | — (no radiographic strata) | EQ-5D/SF-12 trajectories to 5 y | No severity stratification; not comparable to moderate vs severe framework | →/NA | Descriptive trajectories only |
| Treu 2024 | Retrospective comparative cohort | Etiology (dysplasia vs primary OA) | HOOS/OHS/complications, 1 y | Different etiologies, not severity levels | NA | Not a radiographic severity comparison |

*Direction is expressed with Severe as reference: ↑ = Severe better; ↓ = Severe worse; → = no clear difference; NA = not applicable.

**Complications**

**(A)Dislocation**

**(B)Superficial wound infection**

**(C)Periprosthetic fracture**

**Postoperative complications**

Forest plots of postoperative complications comparing patients with moderate versus severe radiographic osteoarthritis after THA.
Complications include (A) dislocation, (B) superficial wound infection, and (C) periprosthetic fracture. Effect estimates are presented as odds ratios (ORs) with 95% confidence intervals, calculated using Mantel–Haenszel fixed-effects models with continuity correction due to sparse events. An OR >1 indicates a higher risk in the moderate osteoarthritis group.

**Other complications**

| Study | Year | Severity definition | Follow-up time (months) | Outcome type | Moderate n / events | Severe n / events |
| --- | --- | --- | --- | --- | --- | --- |
| Neitzke 2024 | 2024 | KL 0–2 vs KL 4 | 12 | Dislocation | n=93, events=2 | n=278, events=3 |
| Neitzke 2024 | 2024 | KL 0–2 vs KL 4 | 12 | Reoperation (any return to OR) | n=93, events=2 | n=278, events=4 |
| Neitzke 2024 | 2024 | KL 0–2 vs KL 4 | 36 | Revision-free survival | 97% revision-free | 99% revision-free |
| Peeters 2024 | 2024 | Tönnis1 vs Tönnis3 | 12 | Dislocation | n=50, events=1 | n=50, events=0 |
| Peeters 2024 | 2024 | Tönnis1 vs Tönnis3 | 12 | Periacetabular muscle envelope | n=50, events=9 | n=50, events=1 |
| Peeters 2024 | 2024 | Tönnis1 vs Tönnis3 | 12 | Psoas tendinopathy | n=50, events=4 | n=50, events=0 |
| Peeters 2024 | 2024 | Tönnis1 vs Tönnis3 | 12 | Greater trochanter pain | n=50, events=6 | n=50, events=1 |
| Peeters 2024 | 2024 | Tönnis1 vs Tönnis3 | 12 | Low back pain | n=50, events=7 | n=50, events=2 |
| Peeters 2024 | 2024 | Tönnis1 vs Tönnis3 | 12 | LFCN compaints | n=50, events=2 | n=50, events=0 |
| Sharrock 2024 | 2024 | KL 0–2 vs 3–4 | 12 | Gruteal tendinopathy | n=70, events=4 | n=200, events=6 |
| Sharrock 2024 | 2024 | KL 0–2 vs 3–4 | 12 | Venous thromboembolism | n=70, events=0 | n=200, events=5 |
| Sharrock 2024 | 2024 | KL 0–2 vs 3–4 | 12 | Psoao impingement | n=70, events=4 | n=200, events=5 |
| Sharrock 2024 | 2024 | KL 0–2 vs 3–4 | 12 | Superficial wound infection | n=70, events=1 | n=200, events=4 |
| Sharrock 2024 | 2024 | KL 0–2 vs 3–4 | 12 | Leg length discrepancy | n=70, events=1 | n=200, events=2 |
| Sharrock 2024 | 2024 | KL 0–2 vs 3–4 | 12 | Periprosthetic fracture | n=70, events=0 | n=200, events=3 |
| Sharrock 2024 | 2024 | KL 0–2 vs 3–4 | 12 | Cellultis | n=70, events=0 | n=200, events=2 |
| Sharrock 2024 | 2024 | KL 0–2 vs 3–4 | 12 | Stiffness | n=70, events=1 | n=200, events=0 |
| Sharrock 2024 | 2024 | KL 0–2 vs 3–4 | 12 | Trochanteric separation | n=70, events=1 | n=200, events=0 |
| Sharrock 2024 | 2024 | KL 0–2 vs 3–4 | 12 | Seroma | n=70, events=1 | n=200, events=0 |
| Sharrock 2024 | 2024 | KL 0–2 vs 3–4 | 12 | Abductor weakness | n=70, events=1 | n=200, events=0 |
| Sharrock 2024 | 2024 | KL 0–2 vs 3–4 | 12 | Intra-operative skin burn | n=70, events=0 | n=200, events=1 |
| Sharrock 2024 | 2024 | KL 0–2 vs 3–4 | 12 | Clicking | n=70, events=0 | n=200, events=1 |
| Sharrock 2024 | 2024 | KL 0–2 vs 3–4 | 12 | Adductor longus avulsion | n=70, events=1 | n=200, events=1 |
| Kashanian 2025 | 2025 | Tönnis2 vs Tönnis3 | 12 | Superficial wound infection | n=260, events=3 | n=420, events=2 |
| Kashanian 2025 | 2025 | Tönnis2 vs Tönnis3 | 12 | Dislocation | n=260, events=1 | n=420, events=2 |
| Kashanian 2025 | 2025 | Tönnis2 vs Tönnis3 | 12 | Periprosthetic fracture | n=260, events=1 | n=420, events=2 |
| Kashanian 2025 | 2025 | Tönnis2 vs Tönnis3 | 12 | Prosthetic joint infection | n=260, events=1 | n=420, events=3 |
| Kashanian 2025 | 2025 | Tönnis2 vs Tönnis3 | 12 | Aseptic loosening | n=260, events=1 | n=420, events=2 |
| Kashanian 2025 | 2025 | Tönnis2 vs Tönnis3 | 12 | Non-orthopaedic complication | n=260, events=0 | n=420, events=2 |

**Sensitivity analysis**

We prespecified one comparison—**Moderate radiographic OA vs Severe OA** (KL/Tönnis 0–2 or 1–2 vs 3–4)—at **12 ± 6 months**. Outcomes were grouped by construct to allow scale-agnostic synthesis:

**Function** (e.g., WOMAC-Function, OHS, HOOS-ADL)

**Pain** (e.g., WOMAC-Pain, VAS)

**Health-related quality of life (HRQoL)** (e.g., EQ-5D, SF-36/VR-12 domains)

Given mixed instruments, the primary meta-analyses used **SMDs under random-effects (IV, DerSimonian-Laird)**. We then ran **instrument-family subgroups** (Function: WOMAC vs OHS vs HOOS; Pain: WOMAC vs non-WOMAC; HRQoL: EQ-5D family vs non-EQ-5D).

Pre-specified sensitivity checks were:

1. **Leave-one-out** analyses within each primary synthesis;
2. **Excluding studies that required median→mean/SD conversion** (Neitzke 2024);
3. **Fixed-effect** re-estimation;
4. **Influential-study removals** (Sharrock 2024, Peeters 2024, Kashanian 2025, Nilsdotter 2001), chosen a priori because of size or leverage.

**Function (instrument subgroups)**

WOMAC-Function only (Kashanian 2025; Nilsdotter 2001):
SMD = −0.05 (95% CI −0.30 to 0.20), I² = 0%, n=92 vs 185.

OHS only (Sharrock 2024; Al-Amiry 2017; Peeters 2024):
SMD = −0.62 (−0.91 to −0.32), I² = 71%, n=330 vs 620.
HOOS-based function (e.g., ADL) only (Peeters 2024; Neitzke 2024):
SMD = −0.31 (−0.97 to 0.34), I² = 87%, n=143 vs 328.
**Primary Function leave-one-out (random-effects, mixed instruments):**
Base model SMD: −0.34 (−0.62 to −0.07), I² = 83%.
Excluding Neitzke 2024 (median→mean/SD):−0.43 (−0.70 to −0.15), I² = 77%.
Excluding Al-Amiry 2017: −0.40 (−0.71 to −0.10), I² = 83%.
Excluding Sharrock 2024: −0.25 (−0.53 to 0.03), I² = 79%.
Excluding Peeters 2024: −0.29 (−0.59 to 0.02), I² = 85%.
Excluding Nilsdotter 2001: −0.39 (−0.69 to −0.10), I² = 85%.

Excluding Kashanian 2025: -0.31 (-0.67 to 0.06), I² = 84%.
Fixed-effect check: −0.37 (−0.47 to −0.27).

**Pain**

WOMAC-Pain only (Gorur 2025; Nilsdotter 2001; Peeters 2024; Neitzke 2024 converted to 0–100 VAS):
SMD = 0.08 (−0.16 to 0.32), I² = 0%, n=130 vs 147.

Non-WOMAC pain only (Neitzke 2024 VAS; Peeters 2024 HOOS-Pain):
SMD = −0.38 (−1.17 to 0.40), I² = 91%, n=143 vs 328.

**Primary Function leave-one-out (random-effects, mixed instruments):**
Base model SMD = −0.14 (−0.51 to 0.22), I² = 79%.
Excluding Neitzke 2024 (median→mean/SD): −0.20 (−0.80 to 0.39), I² = 85%.
Excluding Gorur 2025: −0.23 (−0.78 to 0.33), I² = 84%.

Excluding Nilsdotter 2001: **−0.21** (−0.65 to 0.23), **I² = 85%**.

Excluding Peeters 2024: **0.04** (−0.13 to 0.21). **I² = 0%**.
Fixed-effect check: **−0.08** (−0.24 to 0.07).

**HRQoL**

EQ-5D family (Al-Amiry 2017; Kashanian 2025; Sharrock 2024):
SMD = −0.36 (−0.68 to −0.03), I² = 81%, n=354 vs 714.

Non-EQ-5D HRQoL (Neitzke 2024 VR-12; Peeters 2024 SF-36 mean):
SMD = −0.28 (−0.62 to 0.06), I² = 56%, n=143 vs 328.

**Primary Function leave-one-out (random-effects, mixed instruments):**

Base model SMD = −0.33 (−0.54 to -0.11), I² = 70%.
Excluding Neitzke 2024 (median→mean/SD): −0.38 (−0.65 to -0.12), I² = 74%.
Excluding AI-Amiry 2017: −0.37 (−0.63 to -0.11), I² = 76%.

Excluding Kashanian 2025: −0.37 (−0.66 to -0.08), I² = 75%.

Excluding Peeters 2024: -0.30 (−0.54 to -0.06). I² = 76%.

Excluding Sharrock 2024: −0.21 (−0.33 to -0.09), I² = 0%.
Fixed-effect check: −0.29 (−0.39 to -0.18).

**Supplementary adjusted continuous meta-analysis**

In adjusted continuous analyses restricted to 12-month studies (GIV–MD), outcomes favoured severe OA (MD −4.60 [−9.05, −0.15]; I²=74%). Findings were directionally consistent with models including the 6-month registry; heterogeneity likely reflects scale and cohort differences (OHS vs HOOS-ADL).

**Supplementary adjusted-responder meta-analysis including long-term cohort (Keurentjes 2013)**

We repeated the adjusted responder meta-analysis adding Keurentjes 2013, which reported an HRQoL-based responder outcome (SF-36 Physical Function MCID) at 24–60 months using a mixed-effects logistic model adjusted for age, sex, BMI and Charnley class. For consistency, the published aOR for Severe vs Mild was inverted to represent Mild/Moderate vs Severe and log-transformed; SEs were derived from the 95% CI. The pooled estimate across four studies (Judge 2011; Dowsey 2016; Dowsey 2022; Keurentjes 2013) showed lower adjusted odds of response in less-severe OA: OR 0.52 (95% CI 0.41–0.67), I² = 4% (random-effects).

Gorur reported adjusted odds ratios by domain (WOMAC-Pain/Stiffness/Function, SF-12P/M). The reported 95% CI for the WOMAC-Function responder contained an internal inconsistency (likely a typographical error), which prevented a reliable SE derivation. We therefore did not include Gorur in the adjusted-responder pooling.

**Restricting to studies at low-to-moderate risk of bias**

Restricting to studies at low-to-moderate risk of bias (excluding those rated serious/critical by ROBINS-I) preserved the direction of effects with only modest changes in magnitude.

**Unadjusted responder counts (PASS/SCB/MCID)**

We synthesized dichotomous responder outcomes reported as unadjusted counts using Mantel–Haenszel random-effects models. The direction of effect was set as *Moderate* (KL/Tönnis 0–2 or 1–2) vs *Severe* (3–4); thus OR > 1 favors the Moderate group (higher responder rate).

**PASS (patient acceptable symptom state)** – 2 studies (Neitzke 2024; Kashanian 2025).
Pooled odds ratio 0.85 (95% CI 0.36–1.97; I² = 78%). Effects were inconsistent: Neitzke favored Moderate (OR≈1.39), whereas Kashanian favored Severe (OR≈0.58). Overall, no clear difference between severity groups.

**SCB (substantial clinical benefit)** – 2 studies (Neitzke 2024; Kashanian 2025).
Pooled odds ratio 0.50 (95% CI 0.10–2.43; I² = 91%) with wide confidence intervals and substantial between-study heterogeneity; direction again conflicted across studies. No robust difference was demonstrated.

**MCID (minimal clinically important difference)** – 1 study (Neitzke 2024, HOOS-JR).
Odds ratio 1.03 (95% CI 0.53–2.03); no evidence of a difference in the proportion achieving MCID at ≈12 months.
